# Palbociclib plus aromatase inhibitors in patients with metastatic breast cancer and cardiovascular diseases: real-world effectiveness

**DOI:** 10.1093/oncolo/oyae273

**Published:** 2024-10-17

**Authors:** Adam Brufsky, Xianchen Liu, Benjamin Li, Lynn McRoy, Connie Chen, Doris Makari, Rachel M Layman, Hope S Rugo

**Affiliations:** Division of Hematology/Oncology, Department of Medicine, UPMC Hillman Cancer Center, University of Pittsburgh Medical Center, Pittsburgh, PA 15213, United States; Pfizer Inc, New York, NY 10001, United States; Pfizer Inc, New York, NY 10001, United States; Pfizer Inc, New York, NY 10001, United States; Pfizer Inc, New York, NY 10001, United States; Pfizer Inc, New York, NY 10001, United States; Department of Breast Medical Oncology, The University of Texas MD Anderson Cancer Center, Houston, TX 77030, United States; Division of Hematology/Oncology, Department of Medicine, University of California San Francisco Helen Diller Family Comprehensive Cancer Center, San Francisco, CA 94158, United States

**Keywords:** breast neoplasms, comparative effectiveness research, electronic health records, palbociclib, retrospective studies, cardiovascular diseases

## Abstract

**Background:**

Patients with cardiovascular disease (CVD) comorbidities are often excluded from participating in breast cancer clinical trials. Consequently, data to inform treatment decisions for patients with hormone receptor–positive/human epidermal growth factor receptor 2–negative (HR+/HER2−) metastatic breast cancer (mBC) and CVD are limited.

**Objective:**

We compared the effectiveness of first-line palbociclib plus an aromatase inhibitor (AI) vs an AI alone and evaluated palbociclib treatment patterns in patients with HR+/HER2− mBC and CVD in routine clinical practice.

**Methods:**

Data from the Flatiron Health Analytic Database were captured for patients with HR+/HER2− mBC and CVD who initiated first-line treatment with palbociclib plus an AI or an AI alone between February 2015 and March 2020 (data cutoff: September 30, 2020). Overall survival (OS), real-world progression-free survival (PFS), and treatment patterns were evaluated.

**Results:**

Of the 469 patients with identifiable CVD, 160 received palbociclib plus an AI, and 309 received an AI alone. After stabilized inverse probability treatment weighting, both median OS (40.7 vs 26.5 months; hazard ratio [HR], 0.732 [95% CI, 0.537-0.997]; *P* = .048) and median real-world PFS (20.0 vs 12.5 months; HR, 0.679 [95% CI, 0.512-0.900]; *P* = .007) were significantly prolonged in patients treated with palbociclib plus an AI vs an AI alone. Among patients with a documented palbociclib starting dose, 78.5% started palbociclib at 125 mg/day, and 38.6% experienced dose adjustment.

**Conclusions:**

In this real-world analysis, first-line palbociclib plus an AI was associated with improved effectiveness compared with an AI alone in patients with HR+/HER2– mBC and CVD.

**Trial Registration:**

NCT05361655 (ClinicalTrials.gov)

## Teaser

Patients with cardiovascular disease comorbidities are often excluded from participating in breast cancer clinical trials. This study evaluated palbociclib clinical outcomes in patients with HR+/HER2− metastatic breast cancer and cardiovascular disease in routine clinical practice.

## Graphical Abstract

**Graphical Abstract:**
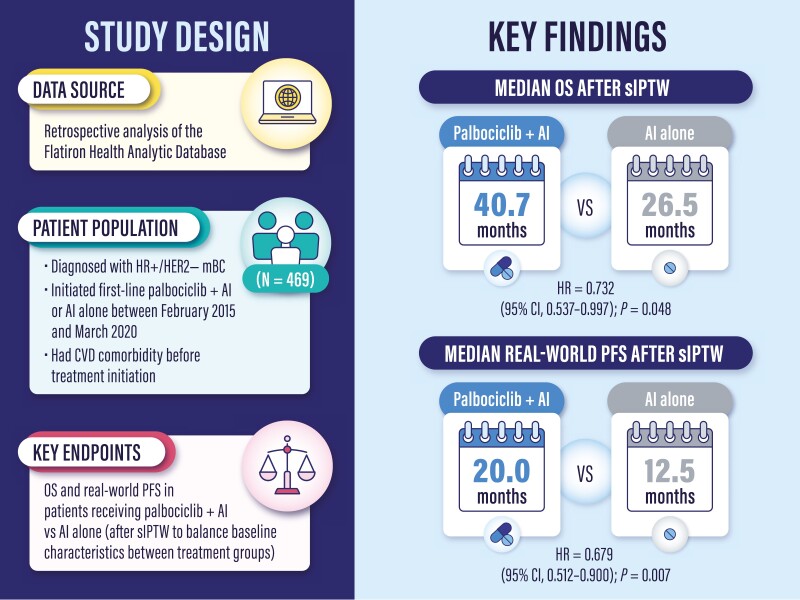

**Implications for practice**

Patients with cardiovascular disease (CVD) are often excluded from breast cancer clinical trials, which can limit the availability of data to inform treatment decision-making for these patients. In this real-world study of patients with HR+/HER2− metastatic breast cancer (mBC) and CVD, first-line palbociclib plus an aromatase inhibitor (AI) was associated with significantly prolonged overall survival and real-world progression-free survival compared with an AI alone. Most patients (78.5%) initiated palbociclib at the recommended dose of 125 mg/day; 38.6% had dose adjustments. This real-world evidence can help inform treatment decisions for patients with HR+/HER2− mBC and CVD in routine practice.

## Introduction

Cardiovascular disease (CVD) comorbidities are prevalent in patients with breast cancer,^1-3^ and an increased incidence of CVD-related mortality has been reported in women with breast cancer compared with those without breast cancer.^4^ Consideration of comorbid CVD is an important element in selecting treatments for patients with breast cancer,^5^ and preexisting CVD comorbidities have been associated with decreased overall survival (OS) and increased mortality in older patients with breast cancer.^6^

Palbociclib, the first-in-class cyclin-dependent kinase 4/6 (CDK4/6) inhibitor, is indicated for the treatment of adult patients with hormone receptor–positive/human epidermal growth factor receptor 2–negative (HR+/HER2−) advanced or metastatic breast cancer (mBC) in combination with an aromatase inhibitor (AI) as initial endocrine-based therapy, or with fulvestrant in patients with disease progression following prior endocrine therapy.^7,8^ Approval of first-line palbociclib plus an AI was supported by the phase 3 PALOMA-2 trial, which showed significant improvement in progression-free survival (PFS) with palbociclib plus letrozole vs placebo plus letrozole among patients with estrogen receptor-positive/HER2− advanced breast cancer.^7,9,10^ Numerical, albeit not statistically significant, improvement in OS was also observed in the palbociclib plus letrozole arm vs the placebo plus letrozole arm.^7^

Patients with preexisting CVD comorbidities, such as heart failure, coronary artery disease, or cerebrovascular disease, are generally underrepresented in oncology randomized clinical trials as they are often excluded.^11,12^ In an evaluation of pivotal cancer clinical trials in the past 2 decades, more than a third excluded patients with certain underlying CVD comorbidities (eg, heart failure, coronary heart disease, arrhythmia, or uncontrolled hypertension), including over 50% of breast cancer trials.^12^ Consequently, data on the efficacy of treatments in patients with comorbid CVD are limited. For example, patients were excluded from PALOMA-2 if they had experienced certain cardiovascular events such as myocardial infarction, atrial fibrillation, symptomatic congestive heart failure, or cerebrovascular accident within 6 months of randomization.^9^ Despite these exclusion criteria, 382 out of 666 patients enrolled (57.4%) in PALOMA-2 had a preexisting vascular or cardiac disorder, the most common of which was hypertension (*n* = 266; 39.9%).^13^ In these patients, the combination of palbociclib and letrozole (*n* = 254) resulted in a median PFS of 30.4 months compared with 14.5 months in the placebo plus letrozole arm (*n* = 128; hazard ratio [HR], 0.51 [95% CI, 0.39-0.66]).^13^ In this analysis, there were no new safety signals observed in patients with preexisting CVD compared to the overall PALOMA-2 population.^13^

Real-world studies can provide evidence for treatment outcomes in patients with mBC seen by physicians in routine clinical practice, including those with CVD.^14^ The P-REALITY X study (NCT05361655) is a real-world, retrospective analysis of electronic health records (EHRs) that compared the effectiveness of first-line palbociclib plus an AI vs an AI alone in patients with HR+/HER2– mBC who were treated in routine clinical practice in the United States.^15^ In P-REALITY X, patients who received palbociclib plus an AI had significantly prolonged real-world PFS and OS compared with those who received an AI alone, both before and after stabilized inverse probability treatment weighting (sIPTW) or propensity score matching.^15^

Prior research on the use and effectiveness of palbociclib plus an AI in patients with HR+/HER2− mBC and CVD is limited. To help inform the use of palbociclib plus an AI in patients with CVD in routine clinical practice, we conducted a subgroup analysis of the P-REALITY X study^15^ and compared the effectiveness of first-line palbociclib plus an AI vs an AI alone in postmenopausal women and men with HR+/HER2− mBC and CVD.

## Methods

### Study design

P-REALITY X was a retrospective, observational, cohort study utilizing data derived from the Flatiron Health Analytic Database. This longitudinal database contains EHRs from >280 cancer clinics, representing >3 million actively treated patients with cancer in the United States. Detailed methods for P-REALITY X have been described previously.^15^ In this subgroup analysis of P-REALITY X, we identified postmenopausal women and men with HR+/HER2− mBC and CVD who initiated first-line treatment with palbociclib plus an AI or an AI alone between February 2015 and March 2020. Patients were assessed from the start of treatment with palbociclib plus an AI or an AI alone until the study data cutoff date (September 30, 2020), death, or last visit, whichever came first. Because P-REALITY X was a retrospective noninterventional study that used anonymized data, it was exempt from institutional review board approval and included a waiver of informed consent.

CVD comorbidities prior to the initiation of palbociclib plus an AI or an AI alone were captured from EHRs in the Flatiron database based on their definitions within the National Cancer Institute (NCI) Comorbidity Index,^16^ and included prior myocardial infarction, congestive heart failure, peripheral vascular diseases, and history of cerebrovascular diseases or events (eg, stroke and transient ischemic attack). The cancer-specific NCI Comorbidity Index was based on the Charlson Comorbidity Index and developed using a cohort of patients with cancer in the Surveillance, Epidemiology, and End Results (SEER)–Medicare database.^16^

### Outcomes

OS was defined as the number of months from initiation of treatment with palbociclib plus an AI or an AI alone until death from any cause. In the Flatiron database, the date of death is a consensus variable across multiple data sources (including the Social Security Death Index, EHRs, and obituary data) and is benchmarked against the National Death Index.^17,18^ Surviving patients were censored at the study cutoff date (September 30, 2020). Real-world PFS was defined as the number of months from initiation of treatment with palbociclib plus an AI or an AI alone until death due to any cause or disease progression, whichever came first.^15,19^ Disease progression was determined based on the treating physician’s clinical assessment or interpretation of radiographic scans or pathology results. If patients survived and did not experience disease progression, those with ≥2 lines of therapy were censored at the start date of the next line of therapy, and those who received only 1 line of therapy were censored at the date of their last visit during the study period. Safety was not assessed in this analysis because adverse event data were not available in the data sources for the P-REALITY X study.

### Statistical analysis

Descriptive statistics were used to describe patient characteristics and treatment patterns, including starting dose, dose adjustments, and subsequent treatments. Effectiveness outcomes were compared both before (unadjusted analysis) and after sIPTW (primary analysis); sIPTW was used to balance baseline demographics and clinical characteristics between treatment groups. The sIPTW method used propensity scores, defined as a patient’s probability of treatment assignment based on measured baseline covariates.^20^ A multivariable binomial logistic regression model generated propensity scores and included the following variables: age group, sex, race or ethnicity, practice type, disease stage at initial diagnosis, Eastern Cooperative Oncology Group performance status, bone disease, visceral disease, interval from initial breast cancer diagnosis to mBC diagnosis, and number of metastatic sites. A standardized difference approach was used to assess the balance of baseline covariates between treatment groups, with a standardized difference of ≥ 0.10 considered to have practical significance.^21^ OS and real-world PFS were summarized using the weighted Kaplan–Meier method and displayed graphically. The weighted Cox proportional hazards model was used to compute HR and corresponding 95% CI for OS and real-world PFS. A multivariable Cox proportional hazards model was used as a sensitivity analysis. Variables used in the estimation of the propensity score were included in the multivariable Cox regression model as covariates.

## Results

### Patients

A total of 469 patients with CVD from the Flatiron database initiated treatment with palbociclib plus an AI (*n* = 160) or an AI alone (*n* = 309) as first-line therapy for HR+/HER2− mBC between February 2015 and March 2020. Median age at mBC diagnosis was 72.0 years in the palbociclib plus an AI group and 77.0 years in the AI-alone group. Approximately 98% of patients were female. CVD comorbidities before treatment initiation (Table 1) included prior myocardial infarction (palbociclib plus an AI group, 25.0%; AI-alone group, 19.7%), congestive heart failure (45.0%; 38.5%), peripheral vascular disease (26.9%; 24.6%), and history of cerebrovascular disease (36.3%; 44.7%). Of the patients in the palbociclib plus an AI cohort, 117 (73.1%), 33 (20.6%), and 10 (6.3%) had 1, 2, and 3 CVDs. Of the patients in the AI-alone cohort, 237 (76.7%), 60 (19.4%), 11 (3.6%), and 1 (0.3%) had 1, 2, 3, and 4 CVDs, respectively. Median follow-up duration was 19.9 months for patients receiving palbociclib plus an AI and 18.9 months for those receiving an AI alone. Compared with patients treated with an AI alone, those treated with palbociclib plus an AI tended to be younger and were more likely to have de novo mBC, ≥ 2 metastatic sites, and lung/liver involvement (**Table 1**). Most baseline covariates used in propensity score estimation were generally well balanced after sIPTW (**Table 1**).

**Table 1.** Patient characteristics.

	Unadjusted total cohort			Cohort after sIPTW		
Characteristics	Palbociclib + AI	AI alone	Standardized difference	Palbociclib + AI	AI alone	Standardized difference
	(*n* = 160)	(*n* = 309)		(*n* = 192)	(*n* = 144)	
Age at mBC diagnosis, years						
Mean (SD)	71.5 (9.0)	74.3 (8.6)	−0.3164	73.3 (9.7)	73.6 (6.0)	−0.0420
Median (IQR)	72.0 (13.0)	77.0 (12.0)		75.0 (12.0)	76.0 (13.0)	
Age at mBC diagnosis^a^						
18-49 years	3 (1.9)	3 (1.0)	0.0764	2 (1.0)	2 (1.4)	−0.0125
50-64 years	29 (18.1)	47 (15.2)	0.0783	28 (14.6)	23 (16.0)	−0.0305
65-74 years	62 (38.8)	80 (25.9)	0.2776	56 (29.2)	43 (29.9)	−0.0220
≥ 75 years	66 (41.3)	179 (57.9)	−0.3383	106 (55.2)	76 (52.8)	0.0450
Sex^a^						
Male	3 (1.9)	6 (1.9)	−0.0049	4 (2.1)	3 (2.1)	−0.0053
Female	157 (98.1)	303 (98.1)		188 (97.9)	141 (97.9)	
Race^a^						
White	103 (64.4)	218 (70.6)	−0.1321	136 (70.8)	100 (69.4)	0.0413
Black	22 (13.8)	40 (12.9)	0.0237	21 (10.9)	18 (12.5)	−0.0468
Other	5 (3.1)	10 (3.2)	−0.0063	5 (2.6)	5 (3.5)	−0.0483
Not documented	30 (18.8)	41 (13.3)	0.1499	29 (15.1)	21 (14.6)	0.0125
Practice type^a^						
Community	150 (93.8)	292 (94.5)	−0.0318	181 (94.3)	136 (94.4)	0.0002
Academic	10 (6.3)	17 (5.5)		11 (5.7)	9 (6.3)	
Disease stage at initial diagnosis^a^						
I	19 (11.9)	54 (17.5)	−0.1588	33 (17.2)	23 (16.0)	0.0422
II	48 (30.0)	78 (25.2)	0.1065	52 (27.1)	38 (26.4)	0.0167
III	21 (13.1)	56 (18.1)	−0.1380	26 (13.5)	23 (16.0)	−0.0748
IV	62 (38.8)	87 (28.2)	0.2260	61 (31.8)	46 (31.9)	−0.0094
Not documented	10 (6.3)	34 (11.0)	−0.1699	20 (10.4)	14 (9.7)	0.0264
ECOG PS^a^						
0	32 (20.0)	63 (20.4)	−0.0097	38 (19.8)	29 (20.1)	−0.0081
1	46 (28.8)	72 (23.3)	0.1244	47 (24.5)	36 (25.0)	0.0005
2, 3, or 4	37 (23.1)	76 (24.6)	−0.0345	51 (26.6)	36 (25.0)	0.0378
Not documented	45 (28.1)	98 (31.7)	−0.0785	56 (29.2)	44 (30.6)	−0.0295
Visceral metastasis^a,b^						
No	110 (68.8)	235 (76.1)	-0.1639	142 (74.0)	106 (73.6)	0.0050
Yes	50 (31.3)	74 (23.9)		50 (26.0)	38 (26.4)	
Bone-only metastasis^a,c^						
No	105 (65.6)	174 (56.3)	0.1918	115 (59.9)	86 (59.7)	
Yes	55 (34.4)	135 (43.7)		77 (40.1)	59 (41.0)	0.0091
Brain metastasis						
No	159 (99.4)	298 (96.4)	0.2061	191 (99.5)	138 (95.8)	0.2605
Yes	1 (0.6)	11 (3.6)		1 (0.5)	6 (4.2)	
Disease-free interval^a,d^						
De novo mBC	62 (38.8)	87 (28.2)	0.2260	61 (31.8)	46 (31.9)	−0.0094
≤1 year	3 (1.9)	12 (3.9)	−0.1203	6 (3.1)	5 (3.5)	−0.0193
>1 to ≤5 years	29 (18.1)	98 (31.7)	−0.3181	42 (21.9)	43 (29.9)	−0.1856
>5 years	65 (40.6)	111 (35.9)	0.0969	83 (43.2)	49 (34.0)	0.1799
Not documented	1 (0.6)	1 (0.3)	0.0439	1 (0.5)	0 (0)	0.0265
Number of metastatic sites^a,e^						
1	80 (50.0)	177 (57.3)	−0.1464	103 (53.6)	79 (54.9)	−0.0175
2	47 (29.4)	52 (16.8)	0.3010	42 (21.9)	31 (21.5)	0.0140
3	18 (11.3)	27 (8.7)	0.0838	16 (8.3)	15 (10.4)	−0.0673
4	6 (3.8)	4 (1.3)	0.1571	5 (2.6)	2 (1.4)	0.0693
≥ 5	3 (1.9)	3 (1.0)	0.0764	3 (1.6)	2 (1.4)	0.0471
Not documented	6 (3.8)	46 (14.9)	−0.3903	23 (12.0)	16 (11.1)	0.0240
Prior myocardial infarction						
No	120 (75.0)	248 (80.3)	−0.1264	150 (78.1)	114 (79.2)	−0.0264
Yes	40 (25.0)	61 (19.7)		42 (21.9)	30 (20.8)	
Congestive heart failure						
No	88 (55.0)	190 (61.5)	−0.1319	108 (56.3)	88 (61.1)	−0.1026
Yes	72 (45.0)	119 (38.5)		84 (43.8)	56 (38.9)	
Peripheral vascular disease						
No	117 (73.1)	233 (75.4)	−0.0522	139 (72.4)	109 (75.7)	−0.0827
Yes	43 (26.9)	76 (24.6)		53 (27.6)	35 (24.3)	
History of cerebrovascular disease						
No	102 (63.8)	171 (55.3)	0.1720	118 (61.5)	79 (54.9)	0.1369
Yes	58 (36.3)	138 (44.7)		74 (38.5)	65 (45.1)	
NCI Comorbidity Index, mean (SD)	1.0 (0.7)	1.0 (0.6)	0.0287	1.0 (0.7)	1.0 (0.4)	−0.0185
Median follow-up duration (IQR), months	19.9 (22.8)	18.9 (27.7)		19.7 (24.0)	18.9 (28.1)	

Data presented as *n* (%), unless specified otherwise.

^a^Covariates used in multivariable Cox proportional hazards models.

^b^Visceral metastasis was defined as metastatic disease in the lung and/or liver; patients could have additional sites of metastasis. No visceral metastasis was defined as no metastatic disease in the lung or liver.

^c^Bone-only metastasis was defined as metastatic disease in the bone only.

^d^Disease-free interval was defined as the interval from initial breast cancer diagnosis to mBC diagnosis.

^e^Multiple metastases at the same site were counted as 1 site. For example, if a patient had 3 bone metastases in the spine, it was counted as 1 site only.

Abbreviations: AI, aromatase inhibitor; ECOG PS, Eastern Cooperative Oncology Group performance status; IQR, interquartile range; mBC, metastatic breast cancer; NCI, National Cancer Institute; SD, standard deviation; sIPTW, stabilized inverse probability treatment weighting.

### Overall survival

**Figure 1.**
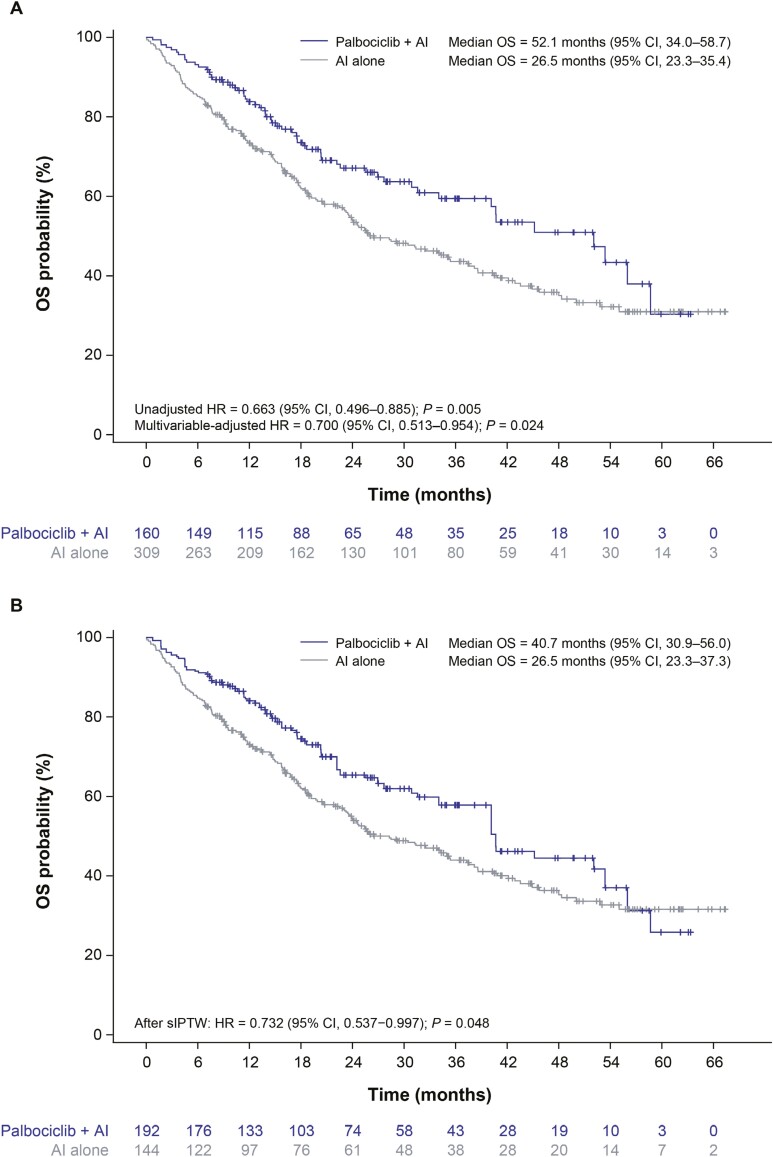
Overall survival. Unadjusted Kaplan–Meier curves of overall survival (A) and Kaplan–Meier curves of overall survival after stabilized inverse probability treatment weighting (B). Abbreviations: AI, aromatase inhibitor; CI, confidence interval; HR, hazard ratio; OS, overall survival; sIPTW, stabilized inverse probability treatment weighting.

In the unadjusted analysis, patients treated with palbociclib plus an AI had significantly longer median OS than patients treated with an AI alone (52.1 months [95% CI, 34.0-58.7] vs 26.5 months [95% CI, 23.3-35.4]; unadjusted HR, 0.663 [95% CI, 0.496-0.885]; *P* = .005; **Figure 1A**). After sIPTW, the median OS was still significantly longer in patients treated with palbociclib plus an AI compared with those treated with an AI alone (40.7 months [95% CI, 30.9-56.0] vs 26.5 months [95% CI, 23.3-37.3]; HR, 0.732 [95% CI, 0.537-0.997]; *P* = .048; **Figure 1B**). Consistent results were observed in the sensitivity analysis (multivariable-adjusted HR, 0.700 [95% CI, 0.513-0.954]; *P* = .024; **Figure 1A**).

### Real-world progression-free survival

**Figure 2.**
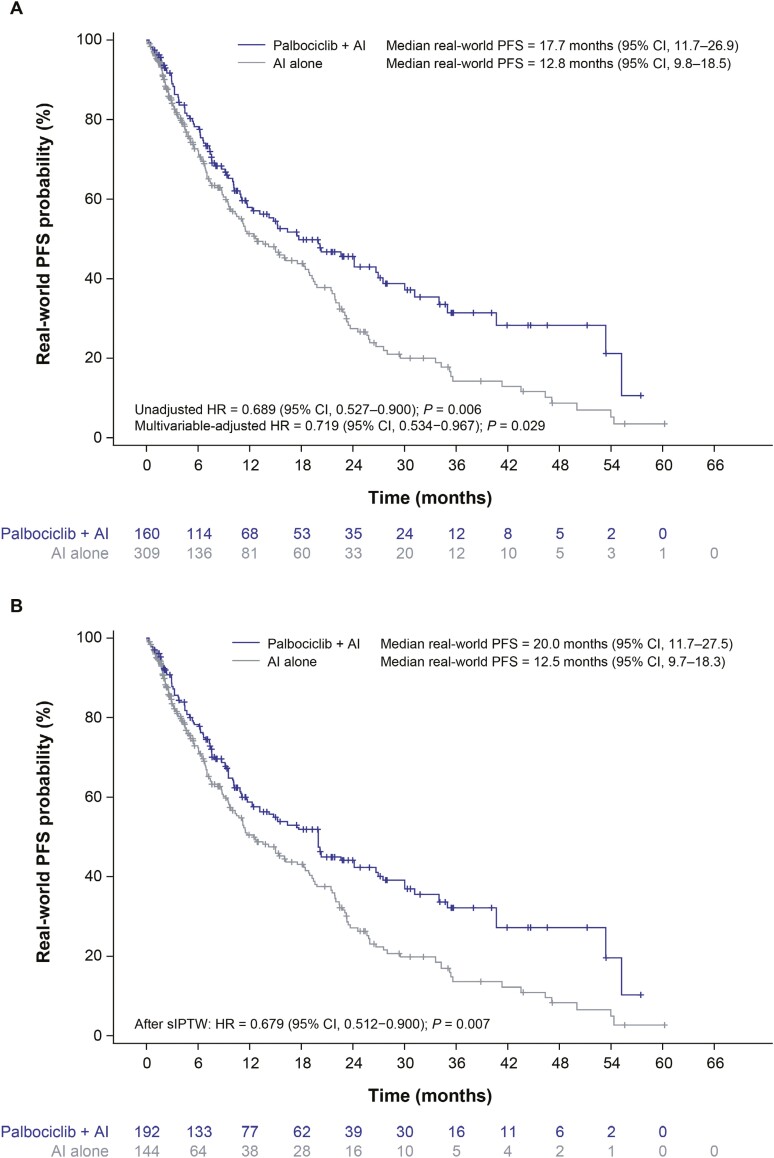
Real-world progression-free survival. Unadjusted Kaplan–Meier curves of real-world progression-free survival (A) and Kaplan–Meier curves of real-world progression-free survival after stabilized inverse probability treatment weighting (B). Abbreviations: AI, aromatase inhibitor; CI, confidence interval; HR, hazard ratio; PFS, progression-free survival; sIPTW, stabilized inverse probability treatment weighting.

In the unadjusted analysis, median real-world PFS was significantly longer in patients treated with palbociclib plus an AI vs an AI alone (17.7 months [95% CI, 11.7-26.9] vs 12.8 months [95% CI, 9.8-18.5]; HR, 0.689 [95% CI, 0.527-0.900]; *P* = .006; **Figure 2A**). After sIPTW, the median real-world PFS was significantly longer among patients treated with palbociclib plus an AI (20.0 months [95% CI, 11.7-27.5]) than for those treated with an AI alone (12.5 months [95% CI, 9.7-18.3]; HR, 0.679 [95% CI, 0.512-0.900]; *P* = .007; **Figure 2B**). The results were similar in the sensitivity analysis (multivariable-adjusted HR, 0.719 [95% CI, 0.534-0.967]; *P* = .029; **Figure 2A**).

### Palbociclib dose adjustment

**Figure 3.**
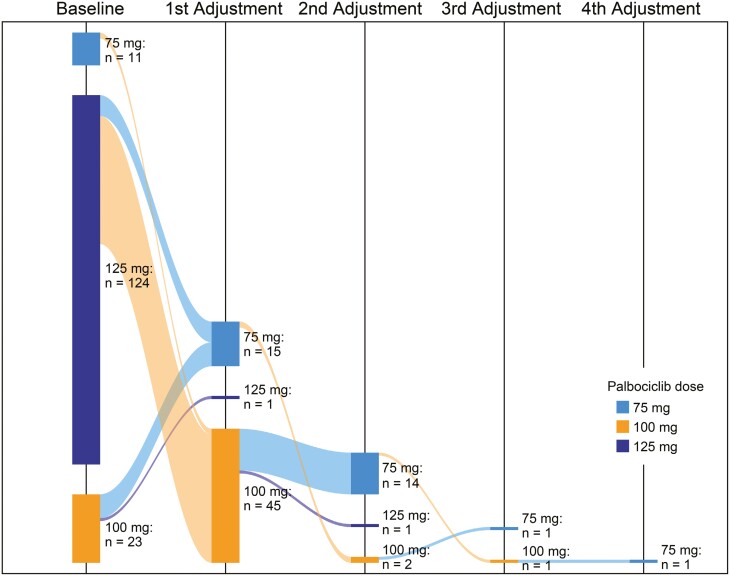
Palbociclib dose adjustments. A Sankey diagram illustrates palbociclib dose adjustments among patients with a documented palbociclib starting dose (*n* = 158).

A total of 158 patients treated with palbociclib plus an AI had a documented palbociclib starting dose, of whom 124 (78.5%) started at 125 mg/day, 23 (14.6%) started at 100 mg/day, and 11 (7.0%) started at 75 mg/day (**Table 2**). In total, 61 of 158 patients (38.6%) experienced dose adjustments. Of the patients who initiated palbociclib at a dose of 125, 100, or 75 mg/day, 50 (40.3%), 9 (39.1%), and 2 (18.2%), respectively, experienced dose adjustments. Of the patients who initiated palbociclib at 100 mg/day and had a dose modification (*n* = 9), 8 had a dose reduction, and 1 had a dose increase. For patients with dose adjustments, the median time to first dose adjustment was 95.5 days among those who initiated palbociclib at 125 mg/day (*n* = 50), 93.0 days among those who started at 100 mg/day (*n* = 9), and 103.5 days among those who started at 75 mg/day (*n* = 2). A Sankey diagram summarizes the dose adjustments in **Figure 3**.

**Table 2.** Palbociclib dose adjustments.

Dose modification	Initial palbociclib dose		
	125 mg/day (*n* = 124)	100 mg/day (*n* = 23)	75 mg/day (*n* = 11)
Any dose change, *n* (%)	50 (40.3)	9 (39.1)	2 (18.2)
125-100 mg/day only	29 (23.4)	—	—
125-100 to 75 mg/day only	13 (10.5)	—	—
125-75 mg/day only	6 (4.8)	—	—
100-75 mg/day only	—	7 (30.4)	—
100-125 mg/day only	—	1 (4.3)	—
75-100 mg/day only	—	—	1 (9.1)
Other change	2 (1.6)	1 (4.3)	1 (9.1)
Median (IQR) number of days to the first dose adjustment among patients with any dose change	95.5 (111.0)	93.0 (182.0)	103.5 (135.0)
Number of dose adjustments (among all patients)			
Median (IQR)	0.0 (1.0)	0.0 (1.0)	0.0 (0.0)
Mean (SD)	0.5 (0.7)	0.4 (0.6)	0.5 (1.2)
Range	0.0 − 3.0	0.0 − 2.0	0.0 − 4.0

Abbreviations: IQR, interquartile range; SD, standard deviation.

### Subsequent treatments

During the follow-up period, 66 of 160 (41.3%) patients in the palbociclib plus an AI group and 184 of 309 (59.5%) patients in the AI-alone group received subsequent second-line anticancer treatment (**Table 3**). Among patients receiving subsequent second-line anticancer treatment in the palbociclib plus an AI group and AI-alone group, respectively, 36.4% and 40.8% received a CDK4/6 inhibitor, and 19.7% and 10.9% received chemotherapy.

**Table 3.** Subsequent second-line anticancer treatments.

Treatments, *n* (%)	Unadjusted total cohort	
	Palbociclib + AI (*n* = 160)	AI alone (*n* = 309)
Any second-line treatment received^a^	66 (41.3)	184 (59.5)
Cyclin-dependent kinase 4/6 inhibitor	24/66 (36.4)	75/184 (40.8)
Chemotherapy	13/66 (19.7)	20/184 (10.9)
Endocrine therapy alone	19/66 (28.8)	81/184 (44.0)
Other anticancer treatments	14/66 (21.2)	23/184 (12.5)

^a^Patients could have received more than 1 category of second-line treatment.

Abbreviation: AI, aromatase inhibitor.

## Discussion

The presence, or risk, of CVD is an important consideration in the treatment of patients with breast cancer, with wide-ranging impacts on treatment selection, toxicities, quality of life, and survival.^5^ For example, CVD comorbidities are associated with decreased OS and increased mortality among patients with breast cancer,^6,22^ and long-term breast cancer survivors have an increased risk of CVD-related mortality compared with women in the general population.^23,24^ Moreover, a large retrospective cohort study of the SEER–Medicare database found that CVD surpasses breast cancer as the leading cause of death in older women with breast cancer, 12 years after the initial breast cancer diagnosis.^25^ However, there is currently limited information on the effectiveness of CDK4/6 inhibitors in patients with HR+/HER2– mBC and CVD. In this retrospective database analysis, we compared the effectiveness of first-line palbociclib plus an AI vs an AI alone and evaluated palbociclib treatment patterns in patients with HR+/HER2− mBC and CVD who were treated in routine clinical practice. We focused our analysis on CVD comorbidities that have been associated with increased mortality or can lead to the exclusion of patients from breast cancer clinical trials, including prior myocardial infarction, congestive heart failure, peripheral vascular diseases, and history of cerebrovascular disease or events.^6^ We found that first-line palbociclib plus an AI, compared with an AI alone, was associated with prolonged OS and real-world PFS in patients with HR+/HER2− mBC and CVD (as defined in this analysis) in a real-world setting.

As expected, patients with CVD treated with palbociclib plus an AI in our study had shorter median OS (40.7 months after sIPTW) than that reported in the general palbociclib plus an AI cohort of P-REALITY X (49.1 months after sIPTW)^15^ or palbociclib plus letrozole cohort of PALOMA-2 (53.8 months).^26^ Similarly, patients receiving an AI alone in our study had shorter median OS (26.5 months after sIPTW) than that observed in the general cohort of patients treated with an AI alone in P-REALITY X (43.2 months after sIPTW)^15^ or placebo plus letrozole cohort of PALOMA-2 (49.8 months).^26^ These findings may reflect a high risk of mortality in our study cohort due to the presence of CVD comorbidities. However, causes of death were not captured in the P-REALITY X study database, and it is possible that other factors besides CVD, such as advanced age, may have contributed to the shorter OS observed in our study cohort despite the use of statistical methods to balance out age between cohorts. Median patient age was higher in the palbociclib plus an AI cohort of our study (75 years after sIPTW) than in the general palbociclib plus an AI cohort of P-REALITY X (70 years after sIPTW)^15^ and the palbociclib plus letrozole cohort of PALOMA-2 (62 years).^26^ Thus, additional research is needed to elucidate the causes of mortality in patients with CVD receiving palbociclib plus an AI vs an AI alone and determine the demographic and clinical factors associated with shorter OS.

The comparative OS benefit of palbociclib plus an AI vs an AI alone in our study of patients with CVD is consistent with findings from prior real-world studies.^15,19,27^ Similar to our study, an OS benefit was observed with palbociclib plus an AI vs an AI alone in the overall population of the P-REALITY X study (after sIPTW: HR, 0.76 [95% CI, 0.65-0.87]; *P* < .001).^15^ Similarly, significant improvement in median OS has been observed with first-line palbociclib plus letrozole vs letrozole alone among patients with HR+/HER2− mBC in a prior Flatiron study (after sIPTW, not reached vs 43.1 months; HR, 0.66 [95% CI, 0.53-0.82]; *P* < .001).^19^ This finding was also supported by a recent study of women aged ≥65 years with HR+/HER2− mBC in the SEER–Medicare database, which found that first-line treatment with a CDK4/6 inhibitor (predominantly palbociclib) plus endocrine therapy was associated with a 41% lower rate of mortality vs endocrine therapy alone (adjusted HR, 0.590 [95% CI, 0.423-0.823]).^27^

The significant improvement in real-world PFS with palbociclib plus an AI compared with an AI alone in our analysis is consistent with PFS results from prior analyses of clinical trial^10,13^ and real-world data.^15,19^ In PALOMA-2, treatment with palbociclib plus letrozole significantly improved PFS compared with placebo plus letrozole in the general study population (27.6 vs 14.5 months; HR, 0.563 [95% CI, 0.461-0.687]; *P* < .001).^10^ A post hoc sub-analysis of patients with preexisting vascular or cardiac disorders (69.6% had hypertension) in PALOMA-2 also reported a PFS benefit, with median PFS of 30.4 months in the palbociclib plus letrozole arm and 14.5 months in the placebo plus letrozole arm (HR, 0.51 [95% CI, 0.39-0.66]).^13^ In the P-REALITY X study, patients in the overall population treated with palbociclib plus an AI had significantly prolonged median real-world PFS after sIPTW compared with those treated with an AI alone (19.3 vs 13.9 months; HR, 0.70 (95% CI, 0.62-0.78); *P* < .001).^15^ Similarly, significant improvement in median real-world PFS has been observed in patients with HR+/HER2− mBC treated with first-line palbociclib plus letrozole vs letrozole alone in a prior Flatiron study (after sIPTW, 20.0 vs 11.9 months; HR, 0.58 [95% CI, 0.49-0.69]; *P* < .001).^19^

In our analysis, we also investigated palbociclib treatment patterns in patients with HR+/HER2– mBC and CVD. Among patients with a documented palbociclib starting dose, 78.5% of patients started palbociclib at the label-recommended dose of 125 mg/day, which was lower than that reported in the general population of P-REALITY X and other real-world studies that evaluated first-line palbociclib plus an AI (ranging from 83.8% to 89.7%).^28-32^ The lowered starting doses of palbociclib observed in our study may reflect heightened safety concerns due to comorbid CVD and concomitant use of medications to manage CVD. In our study, 40.3% of patients who initiated palbociclib at a starting dose of 125 mg/day experienced dose adjustments, generally similar to PALOMA-2 (39.4%) as well as P-REALITY X and other real-world studies (ranging from 29.6% to 37.9%).^10,28,31,32^

We also analyzed subsequent second-line anticancer treatments following first-line palbociclib plus an AI or an AI alone in patients with CVD. Overall, more patients in the AI-alone cohort (59.5%) than in the palbociclib plus an AI cohort (41.3%) proceeded to second-line therapy. Similarly, more patients in the AI-alone cohort vs the palbociclib combination cohort proceeded to second-line therapy in both the overall P-REALITY X population (AI alone, 65.1%; palbociclib plus AI, 48.9%)^15^ and the PALOMA-2 population (placebo plus letrozole, 67.6%; palbociclib plus letrozole, 51.1%).^10^ In our study, following first-line palbociclib plus an AI or an AI alone, use of a CDK4/6 inhibitor in the second-line setting was similarly frequent (36.4% vs 40.8%, respectively). In the palbociclib plus an AI cohort vs the AI-alone cohort, use of second-line chemotherapy was higher (19.7% vs 10.9%), and use of second-line endocrine therapy was lower (28.8% vs 44.0%). Generally similar second-line treatment patterns were observed in the overall P-REALITY X population.^15^ Taken together, these results suggest frequent use of second-line CDK4/6 inhibitors in a real-world setting, irrespective of prior first-line exposure to palbociclib, in both the general population of patients with HR+/HER2− mBC and the subset of patients with CVD comorbidities.

Prior research was limited on the effectiveness of palbociclib plus an AI in patients with HR+/HER2− mBC and CVD (especially in those with severe diseases), and the evidence generated in this analysis can help inform the treatment of this patient subgroup in real-world clinical practice. Strengths of this analysis include the diversity of the patient population in a real-world setting, a follow-up duration of up to 68 months, validated death dates in the Flatiron database, the advanced statistical methodology used to balance patient baseline characteristics, and the consistency in results between primary and sensitivity analyses.

## Study limitations

Interpretation of these results is subject to the typical limitations of a retrospective study design, including a lack of randomization in treatment assignment and the potential for inaccurate or missing data. Furthermore, data from retrospective observational studies cannot be used to infer causal relationships between treatments and patient outcomes. Although standard statistical methodologies (sIPTW and multivariable analyses) were implemented to help balance patient characteristics between treatment groups, the potential for unobserved confounding variables to bias the results could not be statistically controlled. CVD comorbidities prior to the initiation of treatment with palbociclib plus an AI or an AI alone were identified using EHRs in the Flatiron database, which may have underreported the presence of CVD. Disease progression was evaluated by treating clinicians, who did not use standardized criteria nor a predetermined schedule for their assessments. Adverse event data and causes of death were not captured in the P-REALITY X study database. Therefore, future research is needed to confirm these findings and determine the reasons for death in patients with HR+/HER2– mBC with comorbid CVD. Although palbociclib with letrozole at the recommended therapeutic dosing regimen did not show clinically relevant effect on the QT interval,^33^ further research may be needed to assess other cardiovascular risk in patients receiving palbociclib plus an AI. Conclusions drawn from these results may not generalize to other patient populations with HR+/HER2– mBC that were not represented in the Flatiron database or patients with other CVD comorbidities that were not captured in the NCI Comorbidity Index, such as hypertension.

## Conclusion

The results from this comparative analysis of first-line palbociclib plus an AI vs an AI alone indicate that palbociclib plus an AI is associated with prolonged OS and real-world PFS in patients with HR+/HER2– mBC and comorbid CVD in a real-world setting. This evidence can help inform treatment decisions for similar patients in routine practice. Further studies with larger cohorts and comprehensive assessments of comorbidities are needed to enhance understanding of the effectiveness and safety of palbociclib plus an AI in patients with mBC and CVD in routine clinical practice.

## Data Availability

The deidentified data that support this study’s findings from Flatiron Health, Inc., are available upon request subject to a license agreement. Please contact DataAccess@flatiron.com to determine licensing terms and for access to the training, data dictionary, validation, and data sets, or The Flatiron Health Analytic Database at https://flatiron.com/contact/.
